# Efficacy and neuroprotective effects of tetramethylpyrazine in animal models of spinal cord injury: a systematic review, meta-analysis, and exploratory machine-learning study

**DOI:** 10.3389/fphar.2026.1964041

**Published:** 2026-09-30

**Authors:** Shaocong Li, Sichong Qiu, Qiqi Tang, Junxiao Ren, Zewei Zhao, Xiangjin Wang, Xiaohong Fan

**Affiliations:** 1 Department of Orthopaedics, Hospital of Chengdu University of Traditional Chinese Medicine, Chengdu, China; 2 Department of Clinical Medicine, Chengdu University of Traditional Chinese Medicine, Chengdu, China; 3 Department of Integrated Traditional Chinese and Western Medicine, Sichuan Clinical Research Center for Cancer, Sichuan Cancer Hospital & Institute, Sichuan Cancer Center, University of Electronic Science and Technology of China, Chengdu, China; 4 Guangzhou University of Chinese Medicine, Guangzhou, China; 5 Preventive Treatment Center, Shenzhen Bao’an Authentic TCM Therapy Hospital, Shenzhen, China

**Keywords:** animal model, machine learning, meta-analysis, SHAP, spinal cord injury, tetramethylpyrazine, XGBoost

## Abstract

**Background:**

Spinal cord injury (SCI) is a severe neurological disorder characterized by persistent motor dysfunction and complex secondary injury processes. Tetramethylpyrazine (TMP) has shown potential neuroprotective effects in experimental SCI; however, the overall efficacy and factors associated with its therapeutic effects remain unclear. This study systematically evaluated the efficacy of TMP in animal models of SCI and explored potential efficacy-related experimental factors using machine-learning methods.

**Methods:**

PubMed, Embase, Web of Science, CNKI, Wanfang and VIP databases were searched for controlled animal studies investigating TMP treatment in SCI. Hedges’ g and corresponding 95% confidence intervals were calculated using random-effects models. Subgroup analyses, sensitivity analyses and assessments of small-study effects were performed where sufficient data were available. An exploratory XGBoost model combined with SHAP analysis was used to evaluate study-level characteristics potentially associated with BBB effect sizes. Model performance was assessed using repeated 10-fold cross-validation with 20 repetitions, and feature-importance stability was evaluated using 1,000 bootstrap resamples.

**Results:**

Forty-two animal studies were included. Compared with control treatment, TMP significantly improved locomotor recovery, with progressively greater improvements in BBB scores from day 1 to day 28 after injury. TMP also improved inclined-plane performance at most evaluated time points. Biochemical analyses indicated that TMP alleviated oxidative stress by reducing malondialdehyde levels and increasing superoxide dismutase activity. TMP treatment was also associated with reduced concentrations of several pro-inflammatory cytokines and improvements in apoptosis-related outcomes. Repeated 10-fold cross-validation showed poor predictive performance (median R^2^ = −0.314). Bootstrap analyses relatively consistently ranked injury level and sex among the most important features; however, these findings were interpreted as exploratory and hypothesis-generating rather than as validated predictors.

**Conclusion:**

The available preclinical evidence indicates that TMP promotes locomotor recovery and exerts antioxidant, anti-inflammatory and anti-apoptotic effects in animal models of SCI. The exploratory machine-learning analysis did not demonstrate reliable predictive performance, and the observed feature-importance patterns should be regarded as hypothesis-generating. Further standardized animal experiments and well-designed translational studies are required.

**Systematic Review Registration:**

https://www.crd.york.ac.uk/PROSPERO/view/CRD420261459838, identifier CRD420261459838.

## 1 Introduction

Spinal cord injury (SCI) is a devastating neurological condition characterized by persistent impairments in motor, sensory, and autonomic functions, imposing a considerable health and socioeconomic burden on affected individuals, their families, and healthcare systems. In 2019, approximately 20.6 million people worldwide were living with SCI, with nearly 0.9 million new cases occurring during that year (Lu et al., 2025). Traumatic SCI is most commonly caused by falls, traffic accidents, violence, and sports-related injuries, although its epidemiological patterns vary considerably across regions and economic settings (Kumar et al., 2018). Despite improvements in emergency care, surgical decompression, spinal stabilization, and rehabilitation, neurological recovery following SCI remains limited, and no currently available treatment can reliably restore lost spinal cord function (Eli et al., 2021).

The pathological evolution of SCI involves an initial mechanical insult followed by a complex secondary injury cascade. Primary injury directly disrupts neurons, axons, glial cells, and the spinal microvasculature, whereas secondary injury is driven by neuroinflammation, oxidative stress, excitotoxicity, mitochondrial dysfunction, blood–spinal cord barrier disruption, and multiple forms of regulated cell death (Sterner and Sterner, 2022). The post-injury immune response is particularly complex because activated microglia, infiltrating macrophages, lymphocytes, and inflammatory mediators may exert both detrimental and reparative effects depending on their phenotypes and temporal stages (Chio et al., 2021). Moreover, iron accumulation and lipid peroxidation after SCI can induce ferroptosis, thereby contributing to progressive neural tissue loss and functional impairment (Bai et al., 2023). Experimental evidence showing that pharmacological inhibition of ferroptosis can attenuate tissue damage and improve locomotor recovery further supports ferroptosis as a potential therapeutic target in SCI(Ryan et al., 2024). Accordingly, therapeutic agents capable of simultaneously modulating inflammation, oxidative stress, vascular dysfunction, and cell death may offer particular advantages in limiting secondary SCI.

Tetramethylpyrazine (TMP), also known as ligustrazine, is a major bioactive alkaloid associated with *Ligusticum chuanxiong* Hort., a traditional Chinese medicinal herb widely used for disorders related to impaired circulation. Pharmacological evidence indicates that TMP possesses antioxidant, anti-inflammatory, anti-apoptotic, vasoprotective, and microcirculation-improving properties and can modulate mitochondrial homeostasis and several intracellular signaling pathways (Liu et al., 2022). These pleiotropic activities are biologically relevant to several major components of the secondary injury cascade following SCI. In experimental SCI, TMP has been reported to improve locomotor function and attenuate neural tissue damage. For example, TMP promoted angiogenesis and nerve regeneration by regulating the miR-497-5p/EGFL7 axis in rats with SCI (Hao et al., 2023). Other studies suggested that TMP could suppress neuroinflammation through the P2X7R/NLRP3 inflammasome and TNFR1/IκB-α/NF-κB pathways (Fan et al., 2024; Liu et al., 2024b). TMP has also been shown to regulate iron metabolism through the NRF2/antioxidant response element pathway, reduce lipid peroxidation and inhibit ferroptosis following SCI (Tao et al., 2024a). Collectively, these findings suggest that TMP may promote neurological recovery through coordinated regulation of inflammation, oxidative stress, vascular repair, iron metabolism, and regulated cell death.

Nevertheless, the efficacy of TMP and the magnitude of its reported effects vary substantially among experimental studies. Considerable heterogeneity exists in animal characteristics, injury levels, modeling methods, TMP formulations, doses, administration routes, treatment durations, and outcome-assessment time points. A previous systematic review and meta-analysis provided preliminary pooled evidence that TMP improved neurological and motor outcomes in rat models of SCI (Li G et al., 2023). However, conventional subgroup analyses generally examine a limited number of prespecified moderators separately and may not adequately identify nonlinear relationships or interactions among multiple experimental variables. Furthermore, additional evidence has subsequently become available, and a broader synthesis of locomotor, oxidative-stress, inflammatory, apoptosis-related, and other secondary injury outcomes is warranted. Therefore, an updated assessment is required not only to estimate the overall efficacy of TMP but also to investigate the potential mechanisms and experimental conditions associated with variation in its observed effects.

Machine learning (ML) offers a complementary approach for exploring multidimensional and potentially nonlinear relationships within structured preclinical datasets. In particular, eXtreme Gradient Boosting (XGBoost) is a tree-based ensemble algorithm capable of modeling complex relationships among multiple experimental characteristics (Chen and Guestrin, 2016). SHapley Additive exPlanations (SHAP) can quantify the relative contribution of individual variables to the output of tree-based models, thereby facilitating both local and global interpretation of model behavior (Lundberg et al., 2020). However, when the number of included studies is limited, ML-derived feature rankings should not be interpreted as evidence of causality or as an externally validated prediction model. Rather, they should be regarded as exploratory, hypothesis-generating findings that may help identify experimental factors warranting further investigation.

Accordingly, this systematic review and meta-analysis aimed to comprehensively evaluate the effects of TMP on locomotor recovery and secondary injury-related outcomes, including oxidative stress, inflammation, apoptosis, and other pathological indicators, in animal models of SCI. Subgroup and sensitivity analyses were conducted to examine the robustness of the pooled findings and potential sources of heterogeneity. In addition, an exploratory XGBoost model interpreted using SHAP was applied to rank experimental variables potentially associated with the observed treatment effects. By integrating conventional evidence synthesis with exploratory ML-based feature ranking, this study sought to update and extend the previous evidence, clarify the potential neuroprotective mechanisms of TMP, and provide a basis for the standardization of future animal experiments and the translational development of TMP for SCI.

## 2 Methods

### 2.1 Protocol and registration

This systematic review and meta-analysis was conducted and reported in accordance with the Preferred Reporting Items for Systematic Reviews and Meta-Analyses (PRISMA) 2020 statement. The review protocol was prospectively registered in the International Prospective Register of Systematic Reviews (PROSPERO; registration number CRD420261459838).

### 2.2 Search strategy

A comprehensive literature search was conducted in PubMed, Embase, Web of Science, China National Knowledge Infrastructure (CNKI), Wanfang Data, and the VIP Database from their inception to August 2026. Controlled vocabulary terms, including Medical Subject Headings and Emtree terms where applicable, were combined with free-text terms related to tetramethylpyrazine and spinal cord injury. The core search concepts included “tetramethylpyrazine,” “ligustrazine,” “spinal cord injury,” and related terms describing traumatic, compressive, ischemic, and ischemia–reperfusion spinal cord injuries. The search strategy was adapted to the syntax and indexing system of each database. Reference lists of eligible studies and relevant reviews were manually screened to identify additional potentially eligible studies. No restrictions were imposed on publication year.

### 2.3 Eligibility criteria

Controlled *in vivo* animal studies were eligible when they met all of the following criteria: 1.An experimental SCI model was established. Eligible models included traumatic SCI induced by contusion, compression, weight-drop, impactor, clip compression, transection, hemisection or other reproducible mechanical procedures, as well as non-traumatic SCI and spinal cord ischemia–reperfusion injury models. 2.Tetramethylpyrazine, also described as ligustrazine or 2, 3, 5, 6-tetramethylpyrazine, was administered as the principal intervention. 3.The comparator group received vehicle, normal saline, placebo, sham treatment without active pharmacological intervention, or no active treatment. 4.At least one prespecified functional, oxidative-stress, inflammatory, apoptosis-related or other relevant pathological outcome was reported. 5.Sufficient quantitative data were available from the text, tables or figures to calculate an effect estimate.

Studies evaluating different TMP formulations or delivery approaches were eligible when TMP constituted the principal active intervention and an appropriate SCI control group was available. Studies involving combined pharmacological interventions were included only when an eligible comparison allowed the independent contribution of TMP to be determined.

The exclusion criteria were: 1. Clinical studies, *in vitro* studies, *ex vivo* experiments or studies without an experimentally induced SCI model; 2. Reviews, systematic reviews, meta-analyses, editorials, letters, conference abstracts, protocols or other non-original publications; 3. Studies in which TMP was combined with another active drug or intervention and the independent effect of TMP could not be distinguished; 4. Studies without an appropriate SCI control group; 5. Studies reporting insufficient quantitative data for effect-size calculation, including studies for which the required data could not be extracted from figures; and 6. Duplicate or overlapping publications reporting data from the same experimental animals.

When multiple publications reported overlapping experimental cohorts, the report containing the most complete outcome data was retained. Supplementary reports were used only to obtain additional non-overlapping information.

### 2.4 Study selection

All retrieved records were independently screened by two reviewers. After duplicate records were removed, the titles and abstracts were examined to exclude clearly irrelevant publications. Full texts of potentially eligible reports were subsequently assessed against the predefined eligibility criteria. Disagreements were resolved through discussion and consensus or, when necessary, consultation with a third reviewer. Reasons for exclusion at the full-text stage were recorded. The study-selection process was summarized using a PRISMA 2020 flow diagram.

### 2.5 Data extraction and management

Two reviewers independently extracted data using a standardized data-extraction form. Any discrepancies were resolved through discussion and consensus or, when necessary, consultation with a third reviewer. The extracted information included the first author, publication year, animal species and strain, sex, age or body weight, sample size, method of SCI induction, injury severity, spinal cord level, TMP formulation, dose, route and frequency of administration, treatment-initiation time, intervention duration, comparator treatment, follow-up time, and reported outcomes. For continuous outcomes, the mean, measure of dispersion, and number of animals in each group were extracted. When data were reported as standard errors of the mean, they were converted to standard deviations using the corresponding group sample sizes. When numerical data were available only in graphical form, values were digitized using GetData Graph Digitizer version 2.26. Graphs were calibrated against their original coordinate axes, and data extraction was repeated when necessary to minimize measurement error. When a study reported multiple eligible TMP intervention groups sharing a common control group, a single intervention group was selected for each primary pooled analysis to avoid repeated inclusion of the same control animals. The selection considered the prespecified comparison, comparability with other studies, and completeness of outcome reporting. Other eligible intervention groups were retained for subgroup or exploratory analyses when appropriate. Data were extracted at all eligible follow-up time points. Repeated measurements obtained from the same animals were assigned to their corresponding time-specific analyses and were not treated as independent observations within the same pooled estimate. When multiple reports described the same or overlapping experimental cohort, the report providing the most complete data was used as the primary source, while additional reports were used only to obtain non-overlapping information.

### 2.6 Outcome measures

The primary outcome was locomotor recovery assessed using the Basso, Beattie, and Bresnahan (BBB) locomotor rating scale. BBB scores were synthesized separately at 1, 3, 7, 14, 21 and 28 days after SCI. When reported, locomotor recovery assessed using the Basso Mouse Scale (BMS) was analyzed separately from BBB scores. Secondary outcomes included: 1.Inclined-plane test performance; 2.Oxidative-stress indicators, including superoxide dismutase (SOD), malondialdehyde (MDA) and glutathione (GSH); 3.Inflammatory mediators, including tumor necrosis factor-α (TNF-α), interleukin-1β (IL-1β), interleukin-6 (IL-6) and interleukin-10 (IL-10); 4.Apoptosis-related outcomes, including the terminal deoxynucleotidyl transferase-mediated dUTP nick-end labeling (TUNEL)-positive rate and, when sufficient data were available, Bax, Bcl-2 and caspase-3. Higher BBB, BMS and inclined-plane test values, higher SOD activity, higher GSH levels, higher IL-10 levels and higher Bcl-2 expression were interpreted as favorable. Lower MDA, TNF-α, IL-1β and IL-6 levels, lower TUNEL-positive rates, and lower Bax and caspase-3 expression were interpreted as favorable.

### 2.7 Risk-of-bias assessment

Two reviewers independently assessed the risk of bias of the included studies using the SYRCLE risk-of-bias tool. The assessment covered sequence generation, baseline characteristics, allocation concealment, random housing, blinding, random outcome assessment, incomplete outcome data, selective reporting, and other potential sources of bias. Each item was rated as low, high, or unclear risk of bias. Disagreements were resolved through discussion or consultation with a third reviewer. The results were summarized graphically.

### 2.8 Statistical analysis

Data were organized and cross-checked using Review Manager version 5.4. Meta-analyses, subgroup analyses, sensitivity analyses, funnel plots, Galbraith plots, and Egger’s regression tests were performed using Stata SE version 18.0. Continuous outcomes were summarized primarily using Hedges’ g with 95% confidence intervals. Hedges’ g was selected to provide a consistent analytical framework across outcomes measured using different units or methods and to apply a correction for the generally small sample sizes of the included animal studies. Effect sizes were calculated as the mean value in the TMP group minus that in the SCI model-control group. Accordingly, positive effect estimates favored TMP for outcomes in which higher values indicated improvement, such as BBB scores, inclined-plane performance, SOD, GSH, IL-10, and anti-apoptotic markers. Negative effect estimates favored TMP for outcomes in which lower values indicated improvement, such as MDA, pro-inflammatory cytokines, Bax, caspase-3, and the TUNEL-positive rate. Because all studies assessing the primary locomotor outcome used the same 21-point BBB scale, an additional sensitivity analysis was performed using the raw mean difference (MD). This analysis was used to determine whether the primary BBB findings were dependent on standardization by within-study variability and to provide effect estimates that could be interpreted directly in BBB-score points. At each assessment time point, the MD analysis included the same studies and group comparisons as the corresponding Hedges’ g analysis and was conducted using a random-effects restricted maximum-likelihood (REML) model. MD was calculated as the mean BBB score in the TMP group minus that in the SCI model-control group; therefore, positive MD values favored TMP treatment. Statistical heterogeneity was assessed using Cochran’s Q test and the I^2^ statistic. An I^2^ value of 50% or greater was considered to indicate substantial heterogeneity. Because differences were expected in animal characteristics, SCI models, intervention regimens, and outcome measurements, random-effects models were used for the principal analyses. Different outcomes and follow-up time points were analyzed separately. A two-sided P value below 0.05 was considered statistically significant. When sufficient data were available, subgroup analyses of BBB scores were conducted according to reported TMP dose category, administration route, administration schedule (single *versus* repeated administration), SCI model, and spinal cord level. Leave-one-out sensitivity analyses were performed by sequentially excluding each study. Funnel plots were used to explore potential small-study effects, while Galbraith plots were used to identify studies that might contribute disproportionately to heterogeneity. Egger’s regression test was performed only when an analysis included at least 10 effect estimates. The results of these additional analyses were interpreted cautiously because of their limited reliability when only a small number of studies were available.

### 2.9 Exploratory machine-learning analysis

An exploratory XGBoost regression model was used to examine whether selected study-level characteristics were associated with the magnitude of the BBB effect size. The dependent variable was the standardized effect size for the BBB score at the prespecified assessment time point. Candidate predictors included injury level, sex, SCI model, total sample size, formulation, administration route, and control vehicle. Species was initially considered but was excluded because it showed no variation in the analytic dataset. Outcome-assessment time was not included as a predictor because it was structurally linked to the selection of the BBB outcome and could therefore introduce temporal confounding. Dose was also excluded because the administered doses were not quantitatively comparable across different administration routes and formulations, and the available studies did not provide sufficient pharmacokinetic information for conversion to a common exposure metric. Model performance was assessed using repeated 10-fold cross-validation with 20 repetitions. R^2^, root mean squared error (RMSE), and mean absolute error (MAE) were calculated for each repetition. Feature-importance stability was evaluated using 1,000 bootstrap resamples. For each resample, relative XGBoost gain importance and mean absolute TreeSHAP values were calculated, together with the frequency with which each feature ranked among the three most important features. Given the limited number of observations, all machine-learning analyses were considered exploratory and hypothesis-generating.

## 3 Results

### 3.1 Search results

A total of 545 records were identified through database searching, including PubMed (n = 44), Embase (n = 95), Web of Science (n = 61), China National Knowledge Infrastructure (CNKI; n = 90), Wanfang Data (n = 84), and VIP Database (n = 171). After the removal of 292 duplicate records, 253 records remained for title and abstract screening, of which 190 were excluded. A total of 63 reports were sought for retrieval, and one report could not be retrieved. The remaining 62 reports were assessed for eligibility, and 20 were excluded because of unavailable extractable data (n = 10), comparison or combination with other drugs (n = 2), or publication as review articles (n = 8). No reports were excluded solely because they were *in vitro* studies. Ultimately, 42 studies were included in the qualitative synthesis and meta-analysis (Figure 1).

**FIGURE 1:**
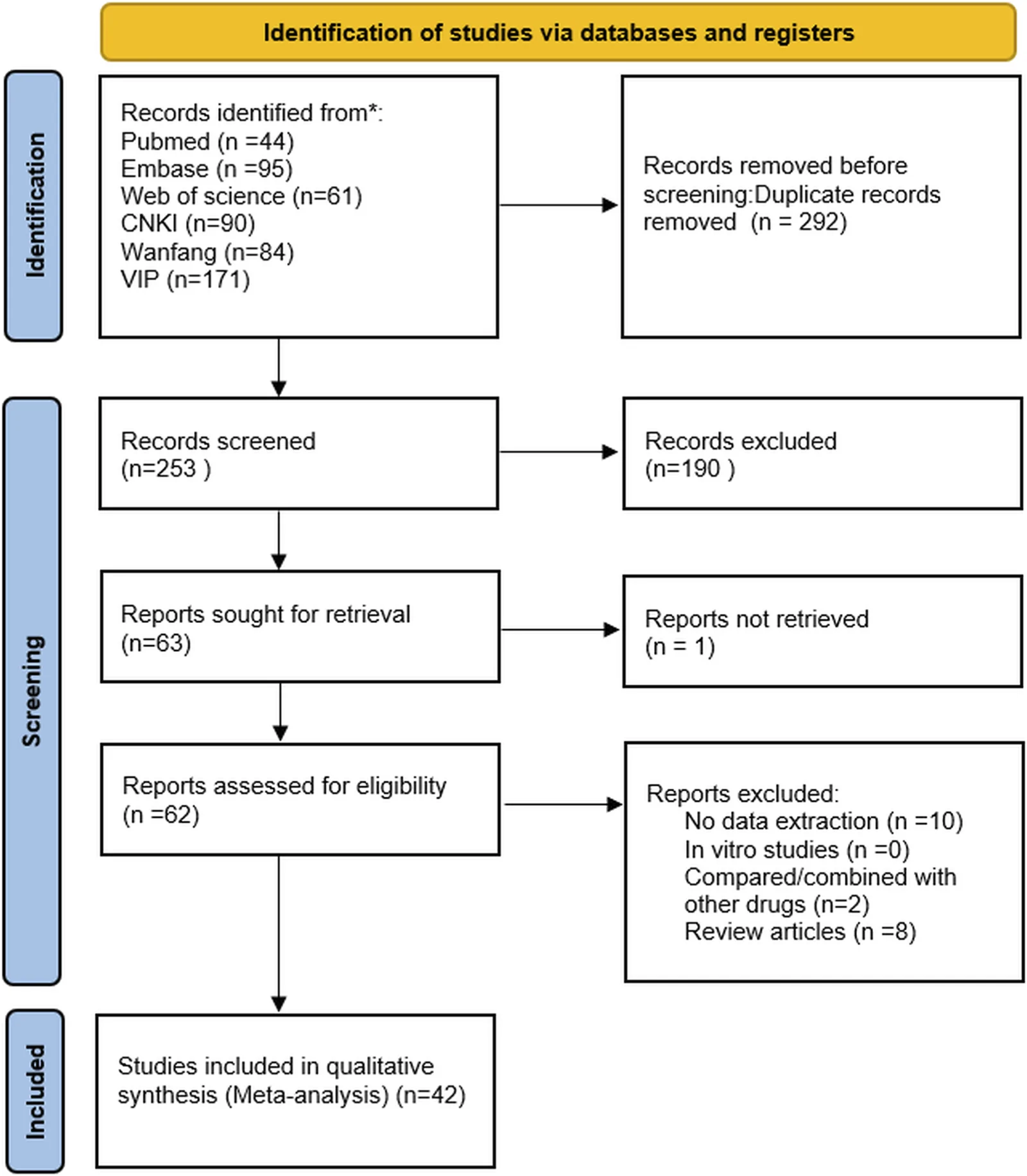
PRISMA 2020 flow diagram of study identification, screening, eligibility assessment, and inclusion.

### 3.2 Characteristics of the included studies

A total of 42 animal studies published between 2002 and 2026 were included (Table 1). Most studies used Sprague–Dawley rats (n = 39), whereas two studies used Wistar rats and one used C57BL/6 mice. Female animals were used in 13 studies, male animals in 11 studies, animals of both sexes in 12 studies, and sex was not clearly reported in six studies.

**TABLE 1:** Characteristics of the included animal studies evaluating the effects of tetramethylpyrazine in experimental spinal cord injury.

Study	Species	Gender	Induction of SCI	Spinal cord	Methods of administration	Intervention IG	Intervention CG	Sample size IG	Sample size CG	Duration of study
Ding et al. (2023)	Wistar rats	Male	Clip-compression	T6–T7	Intraperitoneal injection	Tetramethylpyrazine 20 mg/kg	Equal-volume normal saline SCI control	15	15	20 days
Huo et al. (2025)	SD rats	Female	NYU weight-drop contusion	T9	Intraperitoneal injection	Tetramethylpyrazine 80 mg/kg	Equal-volume normal saline SCI control	10	10	28 days
Fan and Wu (2017)	SD rats	Male	Modified Allen method/ IH impactor contusion	T10	Injection into injured spinal segment	Tetramethylpyrazine 200 mg/kg	Equal-volume normal saline SCI control	5	5	21 days
Wang et al. (2016)	SD rats	NR	Contusive SCI	NR	NR	Tetramethylpyrazine (dose not reported)	Equal-volume normal saline SCI control	8	8	28 days
Hu et al. (2013)	SD rats	Male	Modified Allen weight-drop contusion	T10	Intraperitoneal injection	Tetramethylpyrazine 200 mg/kg	Equal-volume normal saline SCI control	5	5	21 days
Hu et al. (2016)	SD rats	Male	Modified Allen weight-drop contusion	T10	Intraperitoneal injection	Tetramethylpyrazine 200 mg/kg	Equal-volume normal saline SCI control	5	5	28 days
Li et al. (2021)	SD rats	Male	Modified Allen weight-drop contusion	T9-T10	Intravenous injection	Tetramethylpyrazine equivalent 8.4 mg/kg	Equal-volume normal saline SCI control	10	10	28 days
Fan et al. (2024)	SD rats	Male and Female	MASCIS impactor contusion	T10	Intraperitoneal injection	Tetramethylpyrazine 80 mg/kg	Equal-volume normal saline SCI control	18	18	14 days
Huang et al. (2016)	SD rats	Male	Modified Allen weight-drop contusion	T10	Intraperitoneal injection	Tetramethylpyrazine 200 mg/kg	Equal-volume normal saline SCI control	4	4	28 days
Tao et al. (2024c)	SD rats	Female	Contusive SCI	T9	Intraperitoneal injection	Tetramethylpyrazine 80 mg/kg	Equal-volume normal saline SCI control	6	6	42 days
Liu et al. (2024a)	SD rats	Female	Modified Allen/NYU impactor contusion	T9	Intraperitoneal injection	Tetramethylpyrazine 80 mg/kg	Equal-volume normal saline SCI control	9	9	28 days
Liu et al. (2024b)	SD rats	Female	Modified Allen weight-drop	T9	Intraperitoneal injection	Tetramethylpyrazine 200 mg/kg	Equal-volume normal saline SCI control	6	6	28 days
Xiao et al. (2025)	SD rats	Female	Contusive SCI	T10	Intraperitoneal injection	Tetramethylpyrazine 20 mg/kg	Equal-volume normal saline SCI control	6	6	28 days
Hu et al. (2015)	SD rats	Male	Contusive SCI	T10	Intraperitoneal injection	Tetramethylpyrazine 80 mg/kg	Equal-volume normal saline SCI control	60	60	28 days
Bai et al. (2026)	SD rats	Female	Weight-drop contusion	T10	Intraperitoneal injection	Tetramethylpyrazine 80 mg/kg	Physiological saline-treated SCI control	12	6	28 days
Xiao et al. (2012)	SD rats	Male and Female	Contusive SCI	T10	Intraperitoneal injection	Tetramethylpyrazine 200 mg/kg	Equal-volume normal saline SCI control	5	5	28 days
Sun et al. (2005)	SD rats	Male and Female	Contusive SCI	T8	Intraperitoneal injection	Tetramethylpyrazine 200 mg/kg	Equal-volume normal saline SCI control	6	6	28 days
Zhao et al. (2006)	SD rats	Male and Female	Moderate Allen SCI	NR	Intraperitoneal injection	Tetramethylpyrazine 200 mg/kg	Equal-volume normal saline SCI control	6	6	28 days
Qi et al. (2011)	SD rats	Male	Contusive SCI	T10	Intraperitoneal injection	Tetramethylpyrazine 200 mg/kg	Equal-volume normal saline SCI control	6	6	28 days
Shen et al. (2008)	SD rats	Male and Female	Modified Allen	NR	Intraperitoneal injection	Tetramethylpyrazine 200 mg/kg	Equal-volume normal saline SCI control	5	5	28 days
Su (2016)	SD rats	Male and Female	Aneurysm-clip compression	T2	Intraperitoneal injection	Tetramethylpyrazine 200 mg/kg	SCI injury control	10	10	14 days
Zhang et al. (2015)	SD rats	Male and Female	Allen weight-drop	T9	Intraperitoneal injection	Tetramethylpyrazine 200 mg/kg	Equal-volume normal saline SCI control	5	5	14 days
Chen et al. (2017)	SD rats	NR	Modified Allen weight-drop	T10	Intraperitoneal injection	Tetramethylpyrazine 200 mg/kg	Equal-volume normal saline SCI control	39	39	21 days
Fan et al. (2023)	SD rats	Male and Female	NYU impactor contusion	T9–T11	Intraperitoneal injection	Tetramethylpyrazine 80 mg/kg	Equal-volume normal saline SCI control	12	12	28 days
Li et al. (2023)	C57BL/6 mice	Female	Modified Rivlin vascular-clip compression	L1	Intraperitoneal injection	Tetramethylpyrazine 100 mg/kg	Equal-volume normal saline SCI control	15	15	14 days
Wang et al. (2015)	SD rats	Male	Modified Allen weight-drop contusion	T8	Intraperitoneal injection	Tetramethylpyrazine 200 mg/kg	Untreated SCI control	10	10	21 days
Jiang (2023)	SD rats	Female	Complete spinal cord transection with microsurgical double-blade scissors	T9/T10	Intraperitoneal injection	Tetramethylpyrazine 25 mg/kg	Equal-volume normal saline SCI control	6	6	14 days
Wang et al. (2011)	SD rats	Male and Female	Modified Allen weight-drop contusion	T10	Intraperitoneal injection	Tetramethylpyrazine 200 mg/kg	Equal-volume normal saline SCI control	10	10	48 h
Zhou et al. (2010)	SD rats	NR	Modified Allen weight-drop contusion	T9–T11	Intraperitoneal injection	Tetramethylpyrazine 200 mg/kg	Equal-volume normal saline SCI control	5	5	21 days
Zhang et al. (2023)	SD rats	Female	Complete spinal cord transection with microsurgical double-blade scissors	T9/T10	Intraperitoneal injection	Tetramethylpyrazine 200 mg/kg	Equal-volume normal saline SCI control	6	6	14 days
Zhao et al. (2013)	SD rats	Male and Female	Modified Allen weight-drop contusion	T11	Intraperitoneal injection	Tetramethylpyrazine 200 mg/kg	Equal-volume normal saline SCI control	8	8	14 days
Pu et al. (2017)	Wistar rats	NR	Modified Allen/Freeman-Wright weight-drop contusion	T11-T12	Intraperitoneal injection	Tetramethylpyrazine 200 mg/kg	Equal-volume normal saline SCI control	6	6	7 days
Tao et al. (2024a)	SD rats	Female	Modified Allen/NYU weight-drop contusion	T10	Intraperitoneal injection	Tetramethylpyrazine 200 mg/kg	Equal-volume normal saline SCI control	6	6	28 days
Tao et al. (2024b)	SD rats	Female	Modified Allen/NYU weight-drop contusion (10 g from 2.5 cm)	T10	Intraperitoneal injection	Tetramethylpyrazine 200 mg/kg	Equal-volume normal saline SCI control	12	12	28 days
Song et al. (2025)	SD rats	NR	Allen weight-drop traumatic SCI	NR	Intraperitoneal injection	Tetramethylpyrazine 200 mg/kg	Equal-volume saline SCI control	10	10	28 days
Zhang et al. (2020)	SD rats	Male	Modified Allen traumatic SCI	NR	Intraperitoneal injection	Tetramethylpyrazine 100 mg/kg/day	Equal-volume saline SCI control	6	6	14 days
Sun (2002)	SD rats	Male and Female	Allen traumatic SCI	T13-L1	Intraperitoneal injection	Tetramethylpyrazine 200 mg/kg	Saline SCI control	8	8	72 h
Wang and Su (2018)	SD rats	Male	Modified Allen weight-drop contusion	T8	Intraperitoneal injection	Tetramethylpyrazine 200 mg/kg	Equal-dose saline SCI control	12	12	21 days
Jiang et al. (2023)	SD rats	Female	Complete 2 mm spinal cord transection	T9/T10	Local injection into lesion	Tetramethylpyrazine 500 μg/mL	Blank conductive hydrogel SCI control	6	6	28 days
Wu et al. (2016)	SD rats	NR	Allen complete transection injury	T9	Intraperitoneal injection	Tetramethylpyrazine 50 mg/kg	BMSC transplantation alone	15	15	56 days
Wu et al. (2024)	SD rats	NR	Weight-drop contusion	T10	Local injection into lesion	Tetramethylpyrazine-loaded supramolecular conductive hydrogel, 0.2 mg/mL, single 10 μL local injection	Untreated SCI model	15	15	28 days
Deng et al. (2024)	SD rats	Female	Complete 2 mm spinal cord transection	T9/T10	Local injection into lesion	Tetramethylpyrazine-loaded conductive hydrogel, 500 μg/mL	Blank conductive hydrogel	18	18	28 days

Contusion or weight-drop injury was the most common SCI model (n = 31), followed by complete transection models (n = 5) and clip-compression models (n = 3); the specific induction method was insufficiently reported in three studies. SCI was predominantly induced at thoracic levels, particularly between T8 and T11. Intraperitoneal injection was the principal route of TMP administration (n = 36). Four studies administered TMP or TMP-loaded formulations locally into the injured spinal cord, one used intravenous injection, and one did not clearly report the administration route. Study duration ranged from 48 h to 56 days, with 28 days being the most frequently reported duration. Detailed characteristics of the included studies are presented in Table 1.

### 3.3 Quality assessment of the included studies

The risk-of-bias assessment showed that 39 of the 42 included studies had a low risk of bias for random-sequence generation, whereas the remaining three studies were rated as unclear because the randomization method was insufficiently reported. All studies had comparable baseline characteristics and were therefore judged to have a low risk of baseline imbalance. Allocation concealment and random housing were not adequately described in any study and were consequently rated as unclear. For blinding of caregivers and investigators, 38 studies were rated as unclear and four as having a high risk of bias, mainly because the interventions involved visibly different surgical, hydrogel, or cell-transplantation procedures. Random outcome assessment was judged as low risk in 27 studies and unclear in 15 studies, whereas blinding of outcome assessors was rated as low risk in 15 studies and unclear in 27 studies. Regarding incomplete outcome data, 41 studies were rated as low risk and one as high risk. All studies were judged to have a low risk of selective reporting and other potential sources of bias. Overall, the principal methodological limitations were insufficient reporting of allocation concealment, random housing, and blinding procedures. A detailed summary of the risk-of-bias assessment is presented in Figure 2.

**FIGURE 2:**
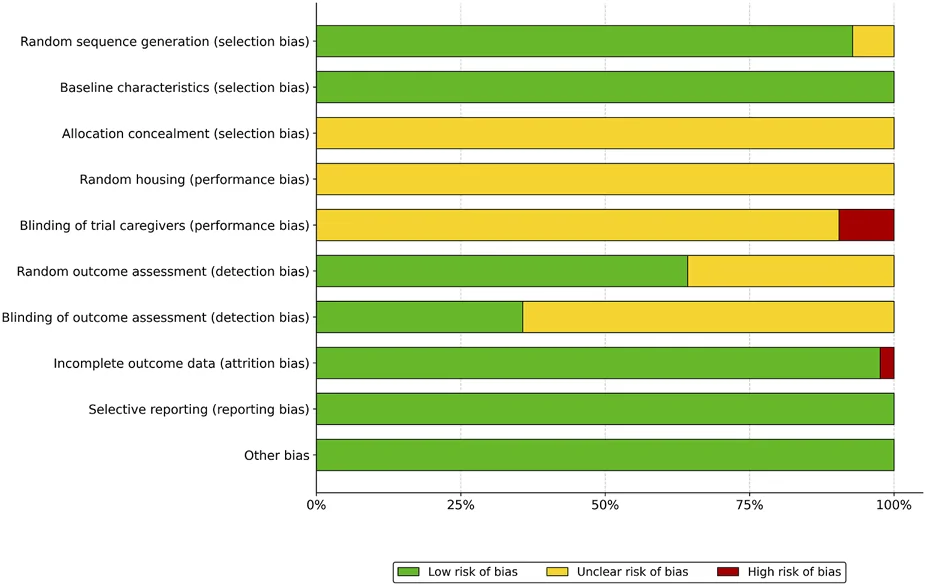
Summary of the risk-of-bias assessment of the included studies using the SYRCLE risk-of-bias tool. Green, low risk of bias; yellow, unclear risk of bias; red, high risk of bias.

### 3.4 BBB scores and subgroup analyses

As shown in Figures 3A–F, BBB scores were significantly higher in the TMP-treated animals than in the controls at all evaluated post-injury time points. At 1 day after SCI, TMP produced a small but significant improvement in BBB scores (Hedges’ g = 0.30, 95% CI 0.10–0.51). The pooled effect subsequently increased at 3 days (Hedges’ g = 0.90, 95% CI 0.56–1.25), 7 days (Hedges’ g = 1.91, 95% CI 1.30–2.53), 14 days (Hedges’ g = 2.85, 95% CI 2.19–3.51), 21 days (Hedges’ g = 3.56, 95% CI 2.86–4.44), and 28 days after SCI (Hedges’ g = 4.43, 95% CI 3.18–5.68). Heterogeneity was low at 1 day but substantial at the later time points. Exploratory subgroup analyses were conducted according to TMP dose and treatment regimen at time points with sufficient data (Supplementary Figure 2). At 3 days, the subgroup receiving 50 mg/kg or less did not show a statistically significant improvement (Hedges’ g = 0.47, 95% CI −0.10 to 1.05), whereas the subgroup receiving 200 mg/kg or more showed a significant effect (Hedges’ g = 1.20, 95% CI 0.69–1.71). At 7 days, significant effects were observed in both the subgroup receiving less than 100 mg/kg (Hedges’ g = 2.28, 95% CI 1.01–3.55) and that receiving 100 mg/kg or more (Hedges’ g = 1.86, 95% CI 1.09–2.63). Repeated administration significantly improved BBB scores (Hedges’ g = 1.70, 95% CI 0.67–2.73), whereas the single-dose subgroup did not reach statistical significance (Hedges’ g = 0.45, 95% CI −0.48 to 1.39). At 14 days, the subgroup receiving less than 100 mg/kg showed a large but non-significant pooled estimate (Hedges’ g = 7.93, 95% CI −0.52 to 16.38), whereas the subgroup receiving 100 mg/kg or more showed a significant effect (Hedges’ g = 2.45, 95% CI 1.69–3.21). The single-dose subgroup was not statistically significant (Hedges’ g = 1.21, 95% CI −0.11 to 2.52), whereas repeated administration significantly improved BBB scores (Hedges’ g = 5.35, 95% CI 0.32–10.38). At 21 days, significant effects were observed in both the subgroup receiving less than 100 mg/kg (Hedges’ g = 5.51, 95% CI 3.22–7.80) and that receiving 100 mg/kg or more (Hedges’ g = 3.15, 95% CI 2.23–4.08). At 28 days, the pooled effects remained significant in both dose subgroups, with estimates of 4.10 (95% CI 2.26–5.95) for less than 100 mg/kg and 5.43 (95% CI 3.07–7.80) for 100 mg/kg or more. Significant effects were also observed for treatment courses of 3 days or shorter (Hedges’ g = 2.46, 95% CI 0.38–4.54) and 7 days or longer (Hedges’ g = 5.49, 95% CI 3.23–7.76). Substantial heterogeneity remained within most subgroups. Because formal tests for differences between subgroups were not performed, these findings were interpreted as exploratory and did not establish a dose-response relationship or the superiority of a particular treatment regimen. The MD-based sensitivity analysis confirmed significantly higher BBB scores following TMP treatment at all assessed time points. The pooled MD increased from 0.14 points at 1 day (95% CI, 0.04–0.24) to 4.67 points at 28 days (95% CI, 3.57–5.77), with intermediate estimates of 0.64, 1.97, 3.19, and 4.49 points at 3, 7, 14, and 21 days, respectively. These findings were consistent with the direction and statistical significance of the primary Hedges’ g analyses, although substantial heterogeneity remained at most time points (Supplementary Figure 7).

**FIGURE 3:**
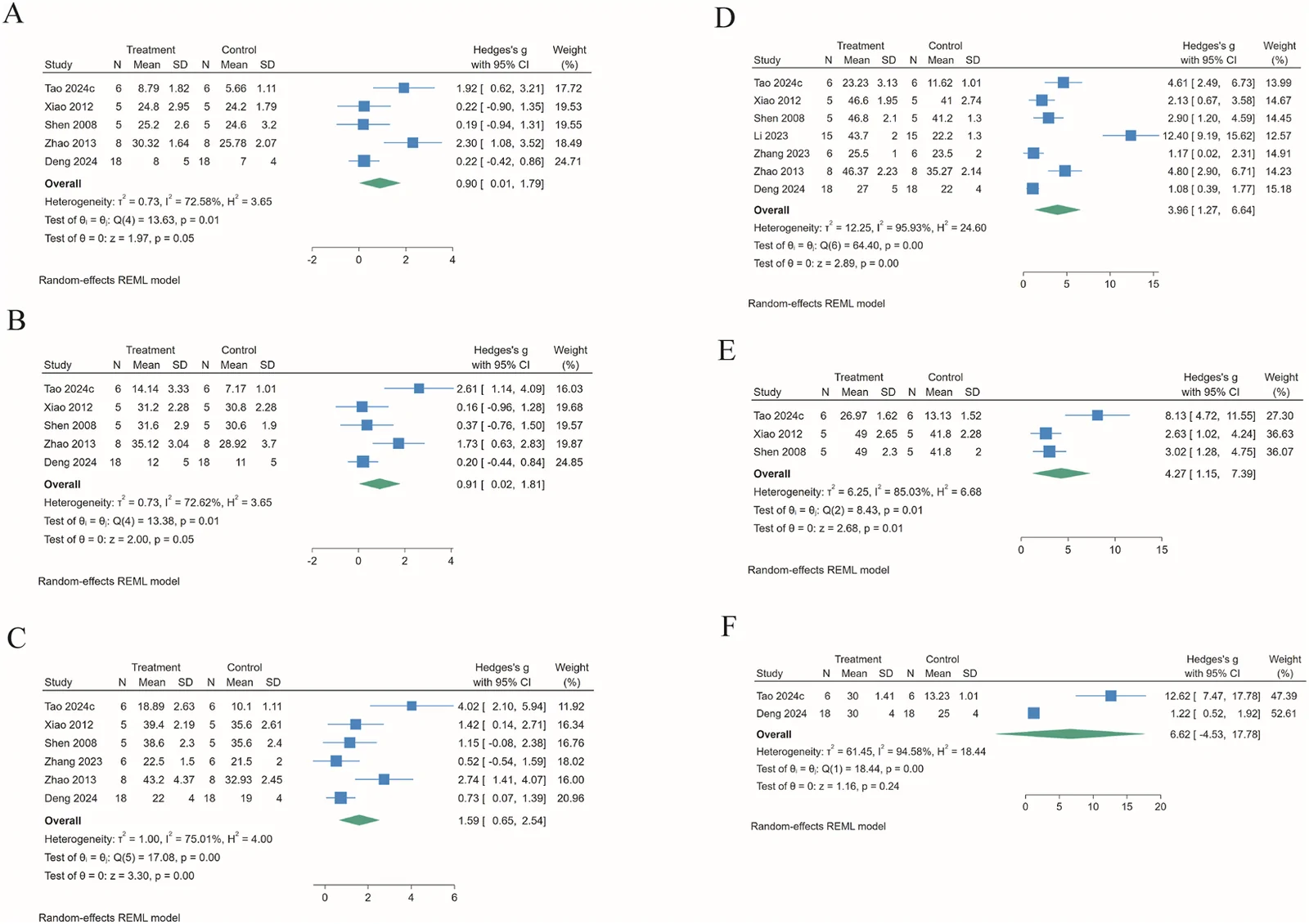
Effects of tetramethylpyrazine on BBB locomotor scores after spinal cord injury. Forest plots showing the effects of tetramethylpyrazine treatment on BBB scores at different post-injury time points: **(A)** 1 day; **(B)** 3 days; **(C)** 7 days; **(D)** 14 days; **(E)** 21 days; and **(F)** 28 days.

### 3.5 Inclined-plane test

As shown in Figures 4A–F, TMP significantly improved inclined-plane performance at 1 day (Hedges’ g = 0.90, 95% CI 0.01–1.79), 3 days (Hedges’ g = 0.91, 95% CI 0.02–1.81), 7 days (Hedges’ g = 1.59, 95% CI 0.65–2.54), 14 days (Hedges’ g = 3.96, 95% CI 1.27–6.64), and 21 days after SCI (Hedges’ g = 4.27, 95% CI 1.15–7.39). At 28 days, the pooled estimate remained positive but was not statistically significant (Hedges’ g = 6.62, 95% CI −4.53 to 17.78). Considerable heterogeneity was present at all time points. Corresponding funnel plots, Galbraith plots and leave-one-out sensitivity analyses are presented in Supplementary Figure 3.

**FIGURE 4:**
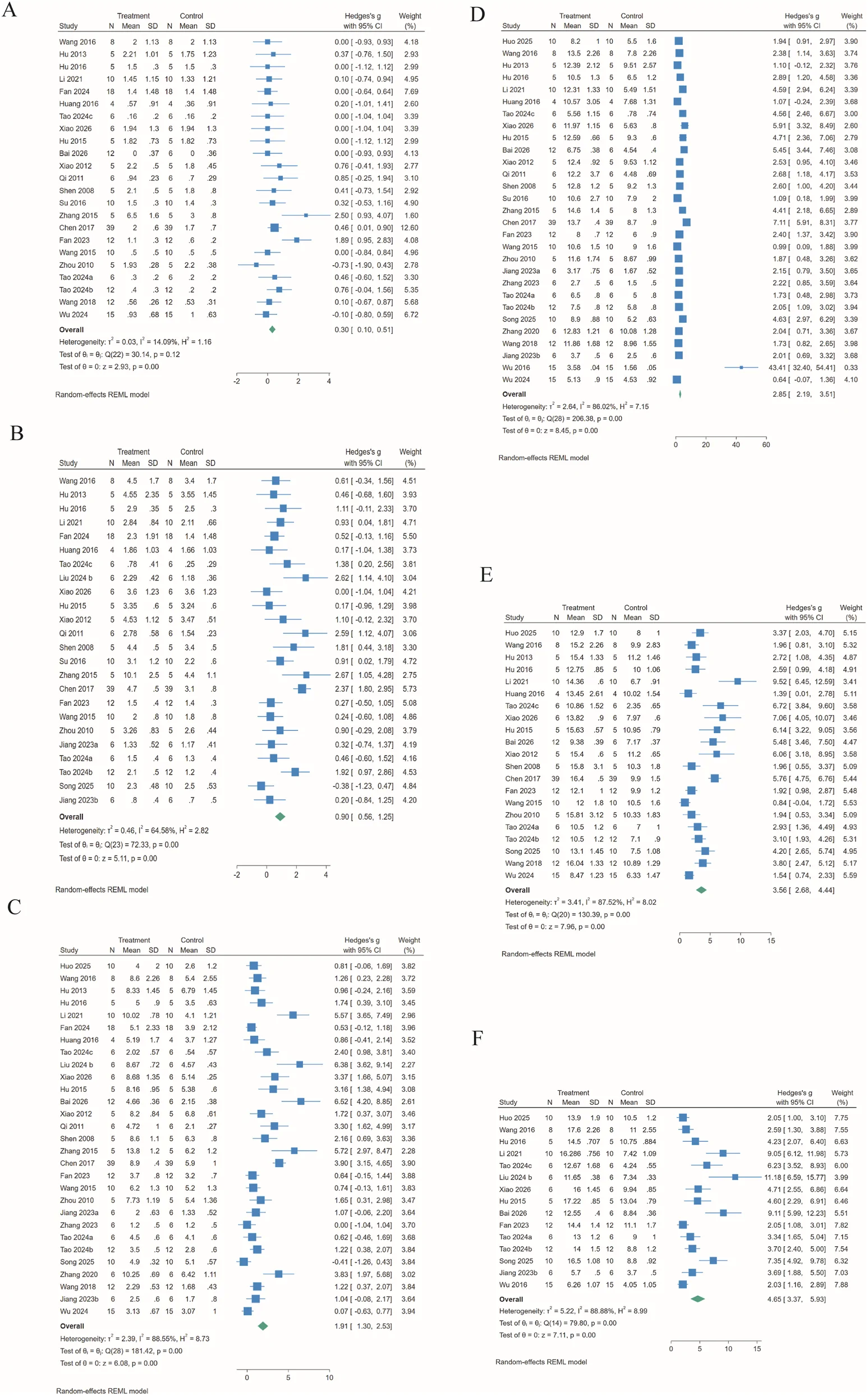
Effects of tetramethylpyrazine on inclined-plane test performance after spinal cord injury. Forest plots show pooled effects at **(A)** 1 day, **(B)** 3 days, **(C)** 7 days, **(D)** 14 days, **(E)** 21 days, and **(F)** 28 days after SCI.

### 3.6 MDA and SOD

As shown in Figures 5A–D, TMP significantly modulated oxidative-stress markers after SCI. MDA levels were significantly lower in the TMP group at 7 days (Hedges’ g = −4.54, 95% CI −7.48 to −1.60) and 28 days (Hedges’ g = −7.14, 95% CI −9.60 to −4.67). SOD activity was significantly higher following TMP treatment at 7 days (Hedges’ g = 8.75, 95% CI 5.83–11.67) and 28 days (Hedges’ g = 8.69, 95% CI 4.41–12.98). Substantial heterogeneity was detected in most analyses. Funnel plots, Galbraith plots and leave-one-out analyses of MDA and SOD are shown in Supplementary Figure 4.

**FIGURE 5:**
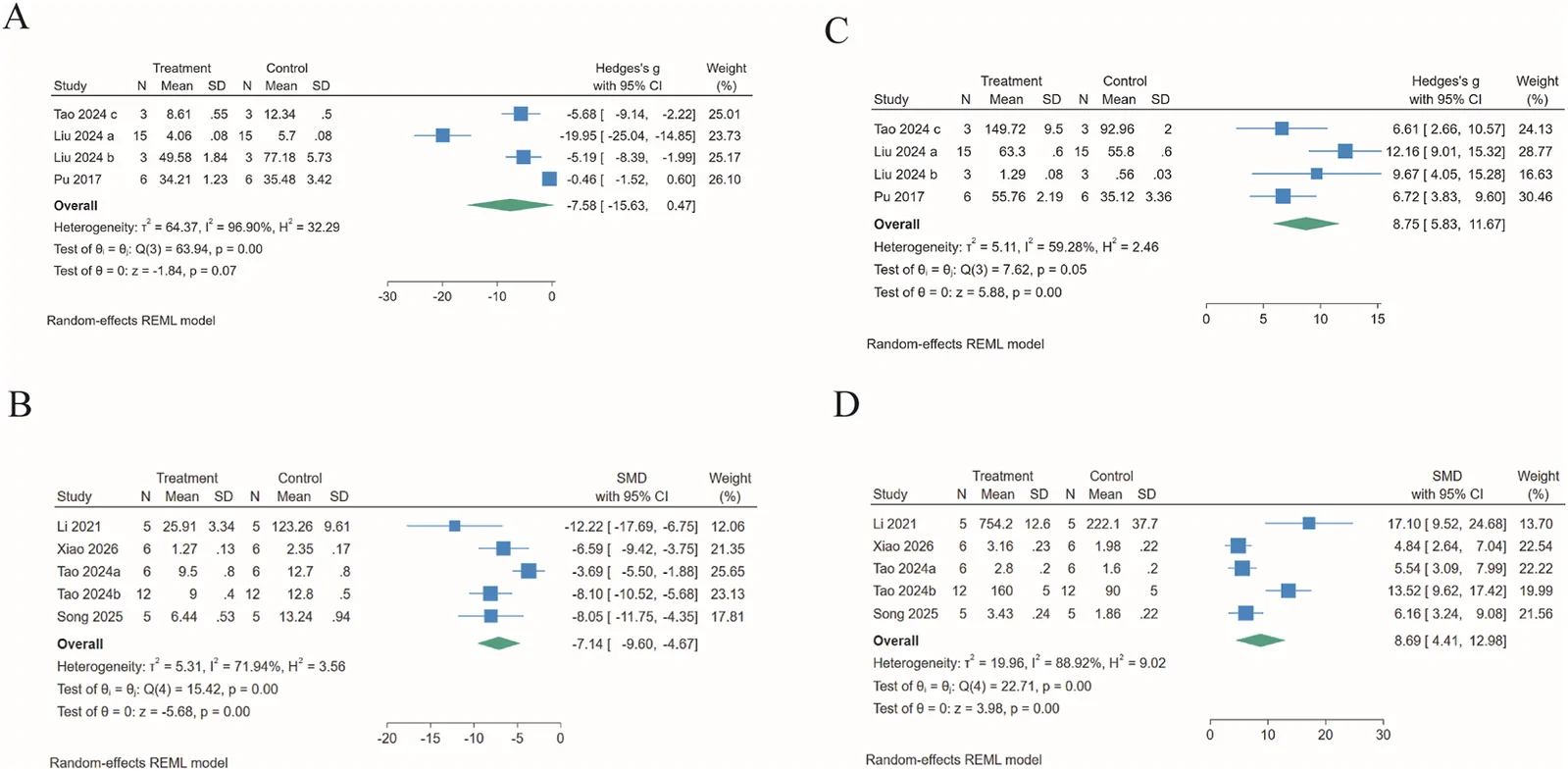
Effects of tetramethylpyrazine on oxidative-stress markers after spinal cord injury. Forest plots show the effects of TMP on MDA levels at **(A)** 7 days and **(B)** 28 days, and SOD activity at **(C)** 7 days and **(D)** 28 days after SCI. MDA, malondialdehyde; SOD, superoxide dismutase.

### 3.7 IL-1β, IL-6, IL-10 and TNF-α

As shown in Figures 6A–D, IL-1β levels did not differ significantly between the TMP and control groups at 1 day (Hedges’ g = −4.18, 95% CI −9.86 to 1.50), 7 days (Hedges’ g = −2.72, 95% CI −6.37 to 0.94), or 28 days after SCI (Hedges’ g = −12.44, 95% CI −28.46 to 3.58). However, IL-1β was significantly reduced at 14 days (Hedges’ g = −2.34, 95% CI −3.60 to −1.08). IL-6 levels were significantly lower in the TMP group at 28 days (Hedges’ g = −5.71, 95% CI −7.07 to −4.35; Figure 6E). IL-10 showed non-significant increasing trends at 7 days (Hedges’ g = 8.86, 95% CI −3.57 to 21.29) and 14 days (Hedges’ g = 15.58, 95% CI −1.34 to 32.49; Figures 6F,G). TNF-α levels were significantly reduced at 7 days (Hedges’ g = −6.36, 95% CI −9.78 to −2.94), 14 days (Hedges’ g = −7.93, 95% CI −15.79 to −0.06), and 28 days after SCI (Hedges’ g = −6.84, 95% CI −10.46 to −3.23; Figures 6H–J). Funnel plots, Galbraith plots and leave-one-out analyses of the inflammatory outcomes are provided in Supplementary Figure 5.

**FIGURE 6:**
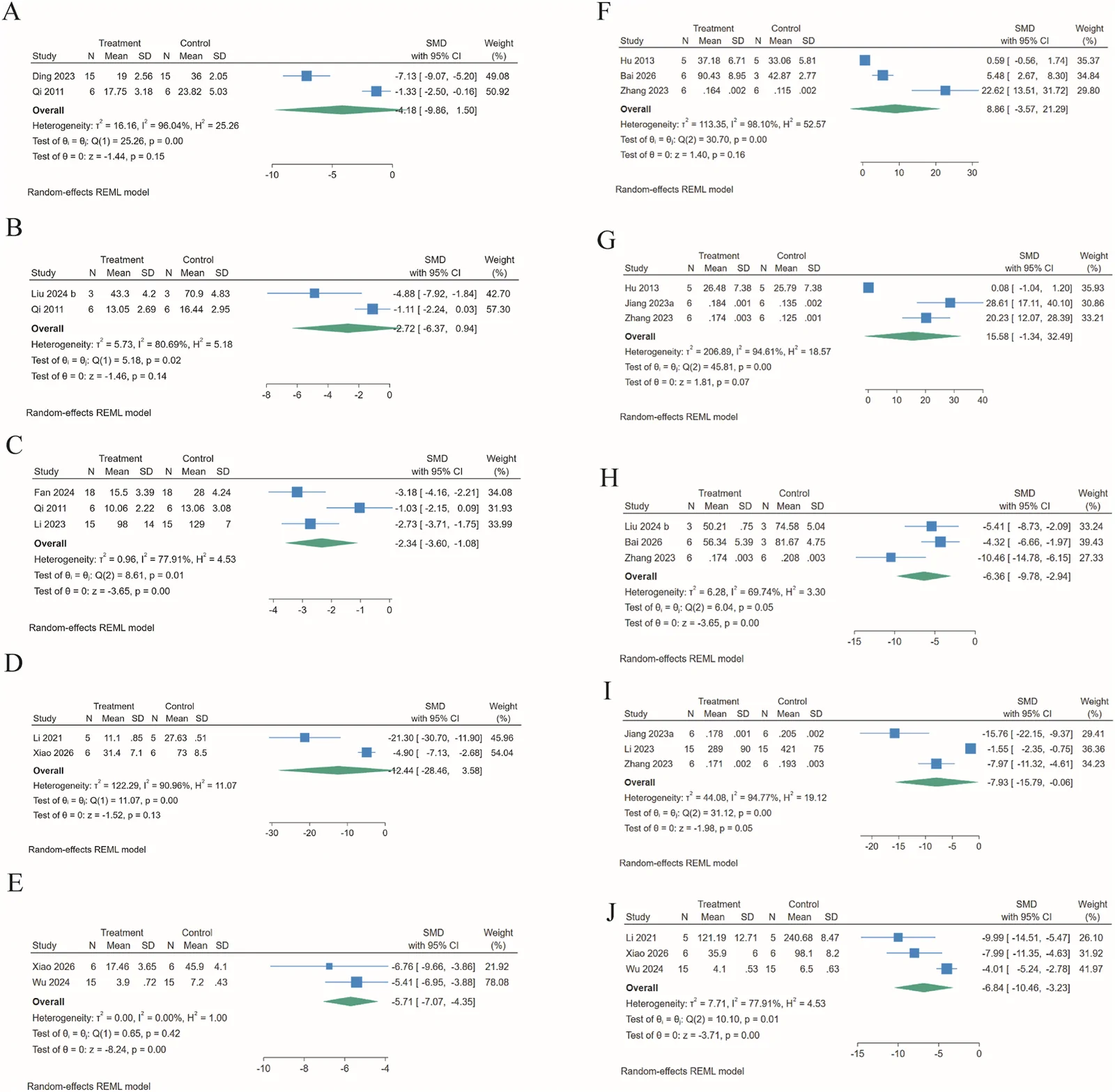
Effects of tetramethylpyrazine on inflammatory mediators after spinal cord injury. Forest plots show the effects of TMP on IL-1β at **(A)** 1 day, **(B)** 7 days, **(C)** 14 days, and **(D)** 28 days; IL-6 at **(E)** 28 days; IL-10 at **(F)** 7 days and **(G)** 14 days; and TNF-α at **(H)** 7 days, **(I)** 14 days, and **(J)** 28 days after SCI.

### 3.8 BAX, BCL-2, caspase-3 and TUNEL

As shown in Figure 7A, TMP significantly reduced BAX levels at 1 day after SCI (Hedges’ g = −3.05, 95% CI −3.92 to −2.17). BCL-2 protein expression was significantly higher in the TMP group at 1 day after SCI (Hedges’ g = 2.78, 95% CI 1.95 to 3.61; Figure 7B). No significant difference in caspase-3 was observed at 1 day (Hedges’ g = −0.36, 95% CI −3.39 to 2.67), whereas caspase-3 was significantly reduced at 7 days (Hedges’ g = −3.33, 95% CI −4.75 to −1.90; Figures 7C,D). The TUNEL-positive rate did not differ significantly at 1 day (Hedges’ g = 0.32, 95% CI −2.23 to 2.86), but was significantly reduced at 3 days (Hedges’ g = −1.01, 95% CI −1.71 to −0.31). At 7 days, the pooled effect was not statistically significant (Hedges’ g = −9.75, 95% CI −21.04 to 1.53; Figures 7E–G). Funnel plots, Galbraith plots and leave-one-out analyses of the apoptosis-related outcomes are shown in Supplementary Figure 6.

**FIGURE 7:**
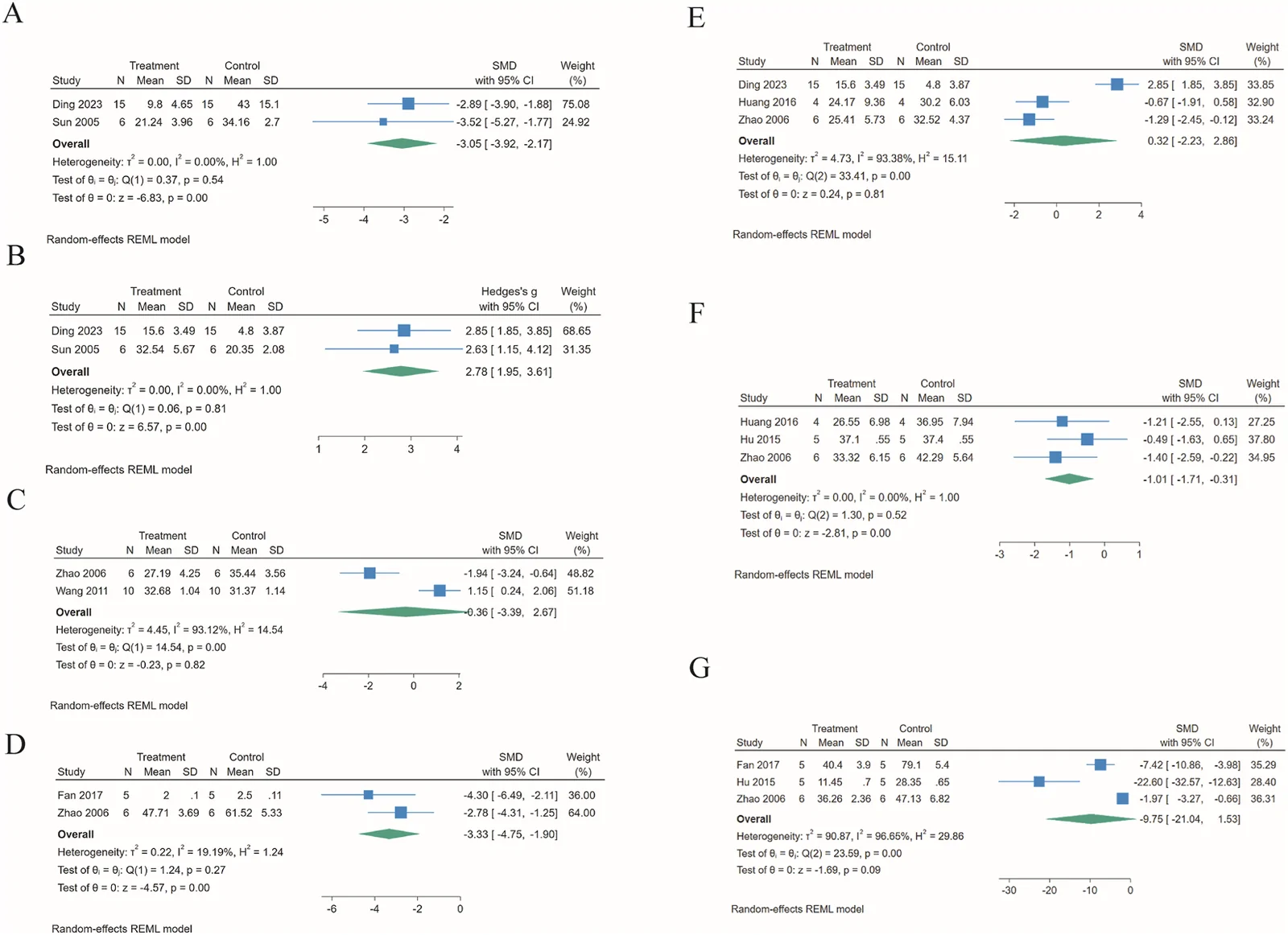
Effects of tetramethylpyrazine on apoptosis-related outcomes after spinal cord injury. Forest plots show the effects of TMP on **(A)** Bax expression at 1 day, **(B)** Bcl-2 expression at 1 day, **(C)** caspase-3 expression at 1 day, **(D)** caspase-3 expression at 7 days, and TUNEL-positive cells at **(E)** 1 day, **(F)** 3 days, and **(G)** 7 days after SCI.

### 3.9 Sensitivity analyses and small-study effects

For BBB scores, the funnel plots, Galbraith plots and leave-one-out sensitivity analyses are presented in Supplementary Figure 1. Sequential exclusion of individual studies did not alter the direction or statistical significance of the pooled BBB effects at any evaluated time point, indicating that the principal findings were relatively robust. However, the Galbraith plots identified several potentially influential studies, particularly at the later time points. Egger’s regression results are summarized in Supplementary Table 1. No evidence of small-study effects was detected at 1 day (P = 0.526) or 3 days (P = 0.826). Significant funnel-plot asymmetry was observed at 7 days (P < 0.001), 14 days (P < 0.001), 21 days (P = 0.003), and 28 days (P < 0.001), suggesting potential small-study effects at the later time points. The inclined-plane results at 1 and 3 days were sensitive to the exclusion of individual studies, whereas the 7-day and 14-day results remained significant in all leave-one-out analyses. The 21-day result became non-significant after exclusion of Shen et al. (2008) (Supplementary Figure 3). The pooled effects for MDA and SOD at 7 and 28 days remained statistically significant after sequential exclusion of each study, supporting the relative robustness of the oxidative-stress findings (Supplementary Figure 4). For inflammatory outcomes, the IL-1β result at 14 days and the TNF-α results at 7 and 28 days remained significant in all leave-one-out analyses. In contrast, the TNF-α result at 14 days and the IL-10 result at 14 days were sensitive to individual studies (Supplementary Figure 5). The 1-day TUNEL result was unstable, and the significant reduction at 3 days became non-significant after exclusion of Zhao et al. (2006). The 7-day TUNEL result remained non-significant in all leave-one-out analyses (Supplementary Figure 6). Because fewer than 10 studies contributed to most secondary-outcome analyses, Egger’s regression tests were not performed for these outcomes. Their funnel and Galbraith plots were interpreted descriptively and were not considered sufficient to confirm or exclude publication bias.

### 3.10 Exploratory machine-learning results

After exclusion of outcome-assessment time and dose from the predictor set, the XGBoost model showed limited out-of-sample predictive performance in repeated 10-fold cross-validation. Across 20 repetitions, the median R^2^ was −0.314 (IQR, −0.348 to −0.293; empirical 95% interval, −0.499 to −0.185), the median RMSE was 3.057 (IQR, 3.033–3.097), and the median MAE was 2.467 (IQR, 2.392–2.524) (Figure 8A). The consistently negative R^2^ values indicate that the model did not outperform prediction based on the mean outcome in held-out observations.Across 1,000 bootstrap resamples, injury level and sex showed the highest median relative XGBoost gain importance, at 30.8% and 23.8%, respectively (Figure 8B). Mean absolute TreeSHAP importance was highest for sex (31.8%) and injury level (30.9%) (Figure 8C). Injury level and sex were ranked among the three most important SHAP features in 91.3% and 89.5% of bootstrap resamples, respectively, followed by sample size in 57.0% and SCI model in 46.3% (Figure 8D). Administration route had a median importance of zero. Because overall predictive performance was poor and uncertainty in the importance estimates remained substantial, these patterns should not be interpreted as validated efficacy-related predictors.

**FIGURE 8:**
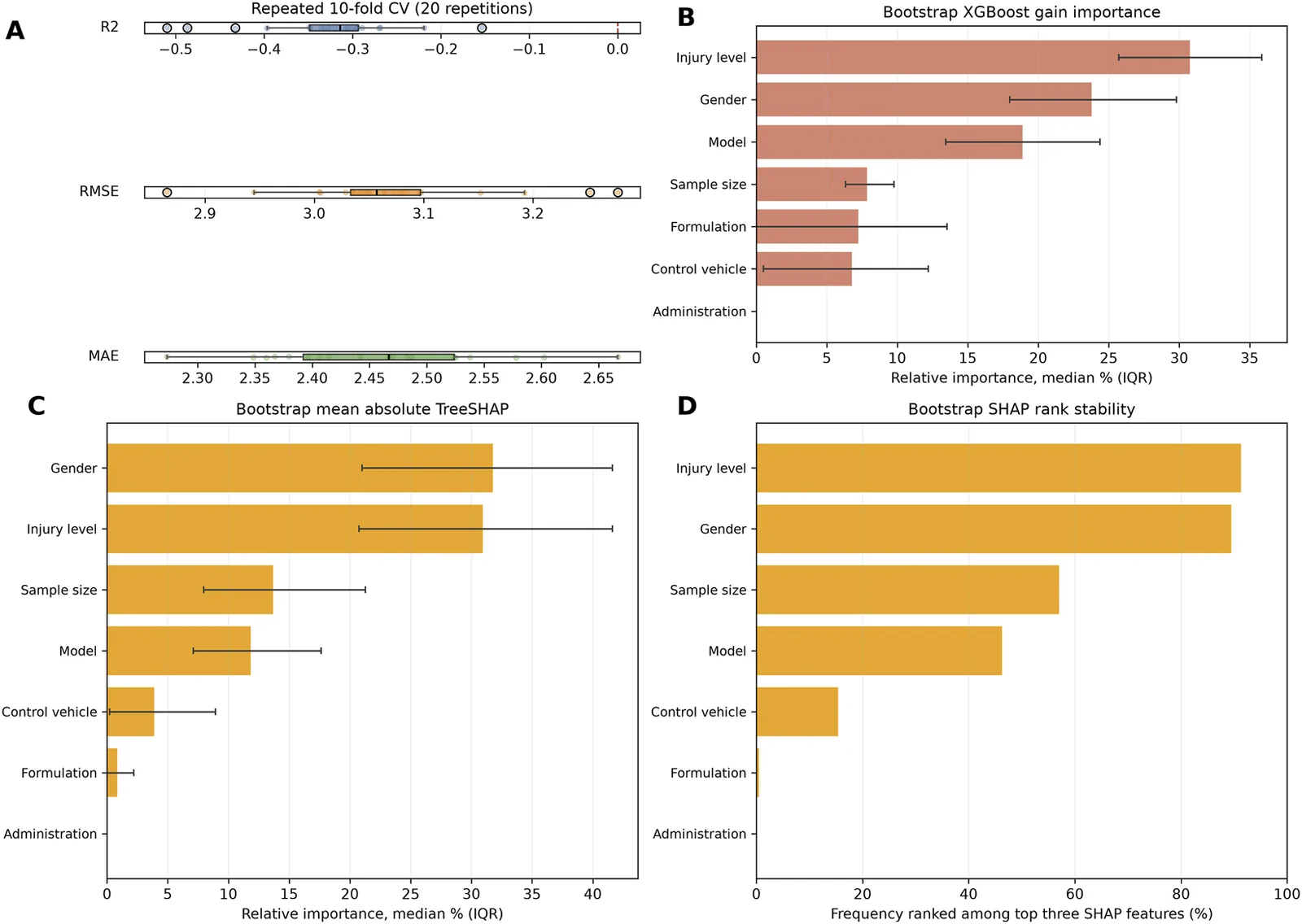
Repeated internal validation and bootstrap stability analysis of the exploratory XGBoost model. **(A)** Distribution of R^2^, RMSE, and MAE across 20 repetitions of 10-fold cross-validation. The dashed red line indicates R^2^ = 0. **(B)** Median relative XGBoost gain importance across 1,000 bootstrap resamples; error bars represent the interquartile range. **(C)** Median relative mean absolute TreeSHAP importance across bootstrap resamples; error bars represent the interquartile range. **(D)** Frequency with which each feature ranked among the three most important features according to mean absolute TreeSHAP values. Outcome-assessment time and dose were excluded from the predictor set because of temporal confounding and lack of cross-study dose comparability, respectively. Feature-importance estimates are exploratory and should not be interpreted as validated efficacy-related predictors.

## 4 Discussion

This systematic review and meta-analysis synthesized evidence from 42 animal studies to evaluate the effects of tetramethylpyrazine (TMP) in experimental spinal cord injury (SCI). The pooled results showed that TMP significantly improved BBB locomotor scores at all evaluated time points from 1 to 28 days after injury. Improvements were also observed in inclined-plane performance at most time points. In addition, TMP reduced MDA levels, increased SOD activity, modulated several inflammatory mediators, and affected apoptosis-related indicators. These findings suggest that TMP may promote neurological recovery after experimental SCI through multiple biological processes involving oxidative stress, neuroinflammation, and programmed cell death. However, substantial heterogeneity, small-study effects at later follow-up points, and methodological limitations of the included studies require cautious interpretation.

The improvement in BBB scores was small at 1 day but became progressively larger at later time points. This temporal pattern may indicate that the beneficial effects of TMP are more closely associated with attenuation of secondary injury and promotion of tissue recovery than with immediate reversal of the primary mechanical injury. The BBB scale is a widely used measure of hindlimb locomotor function in rat SCI models, although its results may be influenced by injury severity, lesion level, animal handling, and assessment procedures (Basso et al., 1995). The inclined-plane results generally supported the BBB findings, particularly between 7 and 21 days. Nevertheless, the 28-day inclined-plane estimate was not statistically significant and showed a very wide confidence interval, indicating imprecision and considerable between-study variability. Therefore, the functional benefits of TMP are supported more consistently by BBB scores than by inclined-plane performance. The MD-based sensitivity analysis was consistent with the primary Hedges’ g analysis, showing significantly higher BBB scores following TMP treatment at all assessed time points. Because MD expresses the treatment effect directly in points on the 21-point BBB scale and does not standardize the effect according to within-group variability, this consistency reduces concern that the observed improvement was solely driven by small within-group standard deviations. Nevertheless, substantial heterogeneity remained at most time points, indicating considerable between-study variation in the absolute magnitude of BBB improvement. Thus, the MD analysis strengthens confidence in the direction of the effect but not in a single precise estimate of its magnitude.

Exploratory subgroup analyses suggested that dose and treatment regimen might contribute to differences in BBB recovery. At 3 days, a significant effect was observed in studies using 200 mg/kg or more, whereas the subgroup receiving 50 mg/kg or less did not reach statistical significance. At 7 and 14 days, repeated administration produced significant improvements, while the corresponding single-administration subgroups were not statistically significant. However, significant effects were observed in both lower- and higher-dose subgroups at several later time points, and substantial heterogeneity remained within most subgroups. Moreover, formal tests for differences between subgroups were not performed. A statistically significant result in one subgroup and a non-significant result in another does not by itself demonstrate a genuine difference between the two subgroups (Sun et al., 2010). These findings should therefore be considered hypothesis-generating and do not establish a dose-response relationship or confirm the superiority of repeated administration. Future studies should directly compare different TMP doses and treatment durations within the same experimental design.

Oxidative stress may represent one of the more consistent biological pathways associated with TMP treatment. TMP significantly decreased MDA and increased SOD activity at both 7 and 28 days, and these findings remained significant in the leave-one-out sensitivity analyses. Following SCI, excessive production of reactive oxygen species, mitochondrial dysfunction, and lipid peroxidation contribute to membrane damage and secondary neuronal and white-matter injury. Ferroptosis, an iron-dependent form of regulated cell death characterized by lipid peroxidation and impairment of antioxidant defense, has also been implicated in SCI progression. Experimental inhibition of ferroptosis has been shown to reduce white-matter injury and improve neurological recovery after SCI (Ge et al., 2022; Huang et al., 2023). The simultaneous reduction in MDA and increase in SOD observed in the present study are therefore compatible with an antioxidant effect of TMP. Nevertheless, these biomarkers are not specific to ferroptosis, and the pooled results cannot independently establish that ferroptosis inhibition is the principal mechanism underlying the functional effects of TMP.

The inflammatory findings were more variable across biomarkers and follow-up periods. TMP significantly reduced TNF-α at 7, 14, and 28 days, while IL-1β was significantly reduced only at 14 days and IL-6 only at 28 days. IL-10 showed increasing trends at 7 and 14 days, but neither estimate was statistically significant. Neuroinflammation after SCI is a dynamic process involving resident microglia, infiltrating monocytes and macrophages, neutrophils, astrocytes, and multiple cytokine networks. Macrophage populations may exert either neurotoxic or reparative effects depending on their phenotype and the post-injury environment (Kigerl et al., 2009). Consequently, the effects of an intervention on inflammatory mediators may vary according to the sampling time, tissue examined, assay method, and injury stage. The present findings provide evidence that TMP may suppress selected pro-inflammatory mediators, particularly TNF-α, but they do not demonstrate uniform suppression of the inflammatory response. The instability of some inflammatory estimates in sensitivity analyses further indicates that these results should be interpreted cautiously.

TMP also affected several apoptosis-related outcomes, although the strength and consistency of the evidence varied across indicators. Bax expression was significantly reduced, whereas BCL-2 protein expression was significantly increased at 1 day after SCI. Caspase-3 expression was significantly reduced at 7 days, whereas no significant difference was observed at 1 day. The TUNEL-positive rate was significantly reduced at 3 days but not at 1 or 7 days, and the 3-day result became non-significant after exclusion of one study. Apoptotic cell death involving neurons and oligodendrocytes may contribute to delayed tissue degeneration after SCI (Crowe et al., 1997). The directionally consistent reduction in the pro-apoptotic marker Bax and increase in the anti-apoptotic protein BCL-2 support a potential anti-apoptotic effect of TMP. Nevertheless, the BCL-2 analysis included only two studies using different protein-detection methods, and the results for caspase-3 and TUNEL were not uniformly significant. Therefore, the apoptosis-related evidence remains preliminary and should be interpreted cautiously.

The exploratory machine-learning analysis was revised to address the limited sample size and the comparability of the candidate predictors. Outcome-assessment time was excluded because it was structurally linked to the selection of the BBB outcome and could introduce temporal confounding. Dose was also excluded because the administered doses could not be reliably compared across different formulations and administration routes in the absence of a common pharmacokinetic exposure metric. The revised analysis therefore evaluated injury level, sex, SCI model, total sample size, formulation, administration route, and control vehicle. Repeated 10-fold cross-validation over 20 repetitions showed consistently negative R^2^ values, with a median R^2^ of −0.314, indicating that the model did not outperform prediction based on the mean outcome in held-out observations. These findings confirm that the current dataset is insufficient for developing a reliable predictive model of the BBB effect size.

Across 1,000 bootstrap resamples, injury level and sex were relatively frequently ranked among the most important features according to both XGBoost gain and mean absolute TreeSHAP values. However, stability of a feature ranking does not establish predictive validity, causality, or treatment-effect modification, particularly when the overall model has poor out-of-sample performance. The observed importance patterns may reflect the limited size and structure of the available dataset, including sparse representation of some categories and correlations among study characteristics. Accordingly, these findings should be regarded only as exploratory and hypothesis-generating signals rather than evidence of validated efficacy-related predictors. Larger datasets with standardized reporting of outcome-assessment time, dose, formulation, administration route, and other experimental characteristics are required before reliable predictors of TMP efficacy can be identified.

The risk-of-bias assessment identified several concerns affecting the certainty and translational relevance of the evidence. Although most studies reported random allocation and comparable baseline characteristics, allocation concealment and random housing were not adequately described. Blinding of caregivers, investigators, and outcome assessors was also incompletely reported in many studies. Insufficient reporting does not necessarily mean that these procedures were not performed, but it prevents reliable assessment of their potential influence on the results. Previous evaluations of animal research have shown that randomization, blinding, sample-size planning, and transparent reporting are essential for reducing exaggerated treatment effects and improving reproducibility (Landis et al., 2012; Macleod et al., 2015). Future studies should follow the ARRIVE 2.0 recommendations and provide explicit information on randomization methods, allocation concealment, blinding, sample-size calculation, excluded animals, and prespecified outcomes (Percie du Sert et al., 2020).

Several additional limitations should be acknowledged. First, substantial heterogeneity was present in most analyses, probably reflecting differences in animal characteristics, injury models, spinal levels, injury severity, TMP dose, formulation, administration route, treatment duration, control conditions, and outcome-measurement methods. Random-effects models and subgroup analyses addressed some of this variability but could not eliminate it. Second, many secondary outcomes were supported by only a small number of studies, resulting in wide confidence intervals and sensitivity to individual experiments. Third, significant funnel-plot asymmetry and Egger’s tests were observed for BBB scores at 7, 14, 21, and 28 days. Publication bias and selective reporting in animal studies may substantially overestimate treatment efficacy (Sena et al., 2010). The increasingly large effect sizes at later time points should therefore not be interpreted solely as evidence of a progressively stronger biological effect. Fourth, the use of standardized effect sizes facilitated pooling across different measurement methods but may yield very large estimates when within-group standard deviations are small. Fifth, most studies used young, otherwise healthy Sprague–Dawley rats, whereas clinical SCI occurs in heterogeneous populations with differences in age, sex, comorbidities, injury mechanisms, and concurrent treatment. Sixth, the machine-learning analysis was based on only 28 study-level observations. Although repeated cross-validation and bootstrap resampling were used for internal assessment, these procedures cannot compensate for the limited information contained in the original dataset (Vabalas et al., 2019). The consistently negative cross-validated R^2^ values indicate poor generalizability, and the feature-importance results should therefore be interpreted solely as exploratory and hypothesis-generating. Finally, no eligible clinical evidence was assessed, and the present results cannot determine the efficacy, optimal dose, pharmacokinetics, or safety of TMP in patients with SCI.

Despite these limitations, this study provides a broad synthesis of the preclinical evidence for TMP in SCI. Compared with the previous meta-analysis (Li G et al., 2023), the present review included a larger and more recent evidence base, evaluated functional outcomes at multiple post-injury time points, synthesized oxidative-stress, inflammatory, and apoptosis-related outcomes, and incorporated subgroup, sensitivity, small-study-effect, and exploratory machine-learning analyses. The consistency of the BBB findings and the robustness of the MDA and SOD results support continued investigation of TMP. Nevertheless, the large pooled effects should be interpreted as evidence of efficacy under experimental conditions rather than as direct estimates of likely clinical benefit.

In conclusion, TMP improved locomotor recovery in animal models of SCI and showed potentially beneficial effects on oxidative stress, selected inflammatory mediators, and some apoptosis-related outcomes. The most consistent evidence was observed for BBB recovery and the regulation of MDA and SOD. However, substantial heterogeneity, possible small-study effects, inconsistent secondary outcomes, and incomplete reporting of bias-control procedures limit confidence in the magnitude of the pooled effects. The machine-learning results were exploratory and did not provide a validated predictive model. Further multicenter animal studies using standardized SCI models, predefined treatment regimens, adequate sample sizes, blinded outcome assessment, and transparent reporting are required before clinical translation can be considered.

## Data Availability

The original contributions presented in the study are included in the article/Supplementary Material, further inquiries can be directed to the corresponding author.

## Supplementary material

SUPPLEMENTARY FIGURE S1 Funnel plots, Galbraith plots, and leave-one-out sensitivity analyses for pooled BBB scores following tetramethylpyrazine treatment at different post-injury time points. **(A–C)** Funnel plot, Galbraith plot, and leave-one-out sensitivity analysis at 3 days, respectively; **(D–F)** corresponding analyses at 7 days; **(G–I)** corresponding analyses at 14 days; **(J–L)** corresponding analyses at 21 days; and **(M–O)** corresponding analyses at 28 days after spinal cord injury.

SUPPLEMENTARY FIGURE S2 Subgroup analyses of BBB scores following tetramethylpyrazine treatment at different post-injury time points. **(A)** ≤50 mg/kg and **(B)** ≥200 mg/kg at 3 days; **(C)** <100 mg/kg, **(D)** ≥100 mg/kg, **(E)** single administration, and **(F)** repeated administration at 7 days; **(G)** <100 mg/kg, **(H)** ≥100 mg/kg, **(I)** single administration, and **(J)** repeated administration at 14 days; **(K)** <100 mg/kg and **(L)** ≥100 mg/kg at 21 days; and **(M)** <100 mg/kg, **(N)** ≥100 mg/kg, **(O)** short-course treatment of ≤3 days, and **(P)** long-course treatment of ≥7 days at 28 days after spinal cord injury.

SUPPLEMENTARY FIGURE S3 Funnel plots, Galbraith plots, and leave-one-out sensitivity analyses for pooled inclined-plane test outcomes following tetramethylpyrazine treatment at different post-injury time points. **(A–C)** Funnel plot, Galbraith plot, and leave-one-out sensitivity analysis at 1 day, respectively; **(D–F)** corresponding analyses at 3 days; **(G–I)** at 7 days; **(J–L)** at 14 days; and **(M–O)** at 21 days after spinal cord injury.

SUPPLEMENTARY FIGURE S4 Funnel plots, Galbraith plots, and leave-one-out sensitivity analyses for oxidative stress-related outcomes following tetramethylpyrazine treatment. **(A–C)** Funnel plot, Galbraith plot, and leave-one-out sensitivity analysis for MDA at 7 days, respectively; **(D–F)** corresponding analyses for MDA at 28 days; **(G–I)** corresponding analyses for SOD at 7 days; and **(J–L)** corresponding analyses for SOD at 28 days after spinal cord injury.

SUPPLEMENTARY FIGURE S5 Funnel plots, Galbraith plots, and leave-one-out sensitivity analyses for inflammatory markers following tetramethylpyrazine treatment. **(A–C)** Funnel plot, Galbraith plot, and leave-one-out sensitivity analysis for IL-1β at 14 days, respectively; **(D–F)** corresponding analyses for IL-10 at 7 days; **(G–I)** for IL-10 at 14 days; **(J–L)** for TNF-α at 7 days; **(M–O)** for TNF-α at 14 days; and **(P–R)** for TNF-α at 28 days after spinal cord injury.

SUPPLEMENTARY FIGURE S6 Funnel plots, Galbraith plots, and leave-one-out sensitivity analyses for TUNEL-positive cells following tetramethylpyrazine treatment. **(A–C)** Funnel plot, Galbraith plot, and leave-one-out sensitivity analysis for TUNEL-positive cells at 1 day, respectively; **(D–F)** corresponding analyses at 3 days; and **(G–I)** corresponding analyses at 7 days after spinal cord injury.

SUPPLEMENTARY FIGURE S7 Sensitivity analysis of BBB locomotor scores using mean differences. Forest plots show the pooled raw mean differences between TMP-treated animals and SCI model controls at **(A)** 1 day, **(B)** 3 days, **(C)** 7 days, **(D)** 14 days, **(E)** 21 days, and **(F)** 28 days after spinal cord injury.
